# Electrolyte Depletion Syndrome due to a 28 cm Rectal Villous Tumor: Successful Endoscopic Resection of One of the Largest Tumors Reported to Date—A Case Report

**DOI:** 10.1002/deo2.70197

**Published:** 2025-08-29

**Authors:** Toshifumi Iida, Hideyuki Chiba, Ai Hirohata, Akimichi Hayashi, Yu Ebisawa, Jun Arimoto, Hiroki Kuwabara, Michiko Nakaoka, Ken Ohata

**Affiliations:** ^1^ Department of Gastroenterology Omori Red Cross Hospital Tokyo Japan; ^2^ Department of Gastrointestinal Endoscopy NTT Medical Center Tokyo Tokyo Japan

**Keywords:** colorectal villous tumor, electrolyte depletion syndrome, endoscopic submucosal dissection, McKittrick–Wheelock syndrome, secretory diarrhea

## Abstract

Electrolyte depletion syndrome (EDS), also known as McKittrick–Wheelock syndrome, is a rare but life‐threatening condition caused by secretory diarrhea from colorectal villous tumors, often accompanied by severe electrolyte imbalances and renal dysfunction. Large, circumferential tumors have traditionally been managed with surgical resection, frequently requiring stoma formation. Recently, endoscopic submucosal dissection (ESD) has emerged as a minimally invasive alternative, although its feasibility for large rectal tumors remains limited. We report a case of EDS caused by a giant circumferential rectal villous tumor measuring approximately 28 cm, successfully treated with ESD. A 58‐year‐old man presented with persistent diarrhea, electrolyte disturbances, and acute kidney injury. Imaging and endoscopy revealed a circumferential villous tumor extending from the anal verge to the rectosigmoid colon, diagnosed as a villous adenoma without malignancy on biopsy. After careful discussion between the departments of gastrointestinal surgery and gastroenterology, ESD under general anesthesia was selected to avoid colectomy and stoma creation. En bloc resection of a 280 × 240 mm tumor was achieved without major complications. Prophylactic steroid injection and systemic steroid administration prevented post‐ESD stricture. Histopathology revealed adenocarcinoma with minimal submucosal invasion (800 µm), no lymphovascular invasion, and negative resection margins, indicating curative resection. At 6‐month follow‐up, no recurrence or stricture was observed. This case highlights the potential of ESD as a definitive and less invasive treatment option for EDS caused by large rectal villous tumors when performed with appropriate therapeutic planning and meticulous postoperative care.

## 1 Introduction

Electrolyte depletion syndrome (EDS), or McKittrick–Wheelock syndrome, is a rare condition caused by secretory diarrhea from colorectal villous tumors, typically presenting with dehydration, electrolyte imbalance, and renal dysfunction that may require urgent treatment [1]. These tumors are often large, and surgical resection has been the standard therapy. Recently, however, sporadic reports have described endoscopic submucosal dissection (ESD) as a minimally invasive alternative [2, 3]. Because of tumor size, ESD is technically demanding, and postoperative management, particularly stricture prevention, remains challenging [4].

We report a case of EDS from a 28‐cm rectal villous adenoma successfully treated by ESD, avoiding colectomy and stoma through an appropriate strategy and prophylactic steroid‐based postoperative management.

## 2 Case Report

A 58‐year‐old man presented with a 6‐month history of persistent diarrhea, which had progressively worsened over the preceding 2 months and was associated with general fatigue and nausea. He was initially evaluated at a local emergency department, where acute enteritis and dehydration were diagnosed. The patient had no prior history of renal dysfunction or electrolyte abnormalities on previous health checkups or other examinations before this episode. Despite hospitalization, his clinical status continued to decline, culminating in acute kidney injury of sufficient severity to warrant consideration of emergent hemodialysis (Table 1). Nevertheless, his condition was ultimately stabilized through aggressive fluid resuscitation and vasopressor support, thereby obviating the need for emergent hemodialysis.

**TABLE 1:** Laboratory findings.

	At the first visit	Prior to treatment	POD1	POW1	POM1	POM6
WBC, ×10^6^/L	22000	10,300	17600	9300	7200	9300
RBC, ×10^12^/L	4.29	3.80	3.53	3.50	3.80	5.46
Hb, g/dL	13.2	11.7	10.9	10.7	11.9	15.4
Hct, %	35.2	35.8	34.6	32.8	36	47.5
Plt, ×10^9^/L	41.5	31.8	32.4	53.4	33.6	35.8
ALP, IU/L	45	50	42	62	103	91
AST, IU/L	83	50	66	33	39	35
ALT, IU/L	77	39	38	39	48	34
LDH, IU/L	148	197	220	187	225	164
T‐bil, mg/dL	1.0	0.7	0.8	0.7	0.9	0.3
G‐GTP, IU/L	98	91	67	132	113	53
TP, g/dL	9.4	6.6	5.5	6.4	6.6	7.2
ALB, g/dL	4.4	3.9	3.1	3.5	3.3	4.1
UA, mg.dL	20.4	—	—	—	—	6.9
BUN, mg/dL	152	22.8	14.6	12.6	19.2	10.6
Cre, mg/dL	7.29	1.29	1.1	0.85	0.73	0.83
AMY, IU/L	53	50	50	74	75	41
CK, U/L	2603	66	3263	186	27	44
Na, mEq/L	123	136	143	140	138	144
K, mEq/L	2.7	4.2	4.8	4.5	4.6	4.2
Cl, mEq/L	80	104	112	103	103	106
CRP, mg/dL	0.5	0.51	5.96	0.43	0.35	0.33
Glucose, mg/dL	154	81	112	82	108	90
HbA1c, %	6.7	6.7	—	—	6.3	6.7
TSH, μIU/mL	4.4	—	—	—	—	1.99
FT3, pg/mL	3.13	—	—	—	—	2.32
FT4, ng/dL	2.17	—	—	—	—	1.94
CEA, ng/mL	6.6	—	—	—	—	5.1

POD: Postoperative Day, POM: Postoperative Month, POW: Postoperative Week.

**FIGURE 1:**
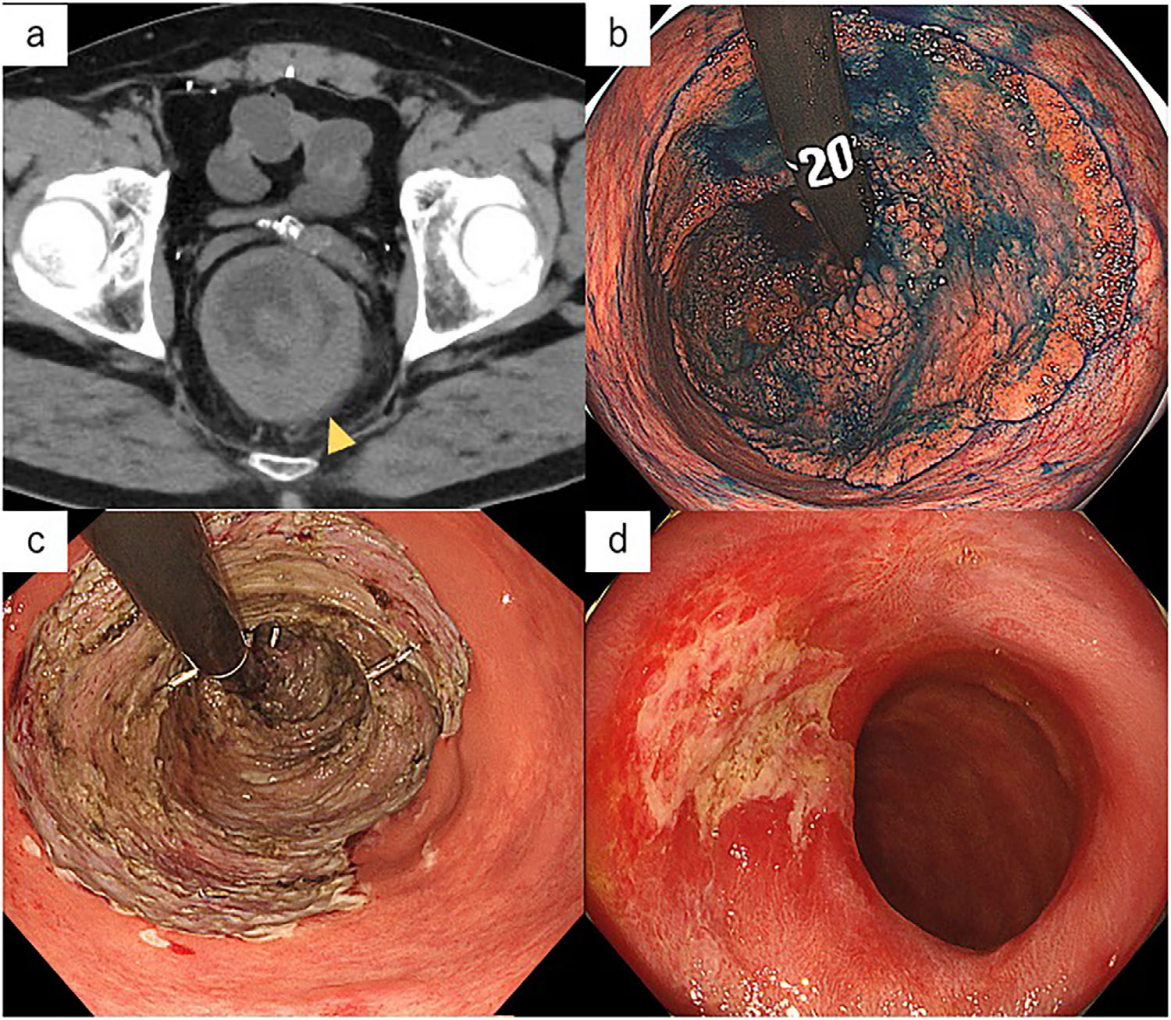
Computed tomography (CT) image and endoscopic images. (a) Pre‐treatment CT image (Axial section), (b) Indigo carmine dye spraying, (c) immediately after treatment, and (d) 6 months after treatment.

Abdominal computed tomography (CT), performed to investigate the etiology of the patient's diarrhea, revealed irregular, circumferential thickening of the rectal wall. No ascites, lymphadenopathy involving the iliac or para‐aortic regions, or distant metastases were observed. Furthermore, there was no evidence of bowel obstruction (Figure 1).

Colonoscopy demonstrated a circumferential villous tumor exceeding 19 cm in length, extending from the anal verge to the rectosigmoid colon (Figure 1). The tumor surface was covered with abundant mucus and exhibited a characteristic villous architecture. Mucosal elevation and erythema were observed above the lower Houston valve and the middle Houston valve. Due to the clinical suspicion of malignancy, an endoscopic biopsy was undertaken, which confirmed the diagnosis of a villous adenoma without evidence of malignant transformation. Given the profuse diarrhea, substantial fluid and electrolyte depletion, and the clinical characteristics of the tumor, including its villous morphology and considerable size, a diagnosis of EDS was established.

Although his vital signs remained stable, his urine output reached 1500 mL per day, while his stool output amounted to 2500 g per day. The patient required daily intravenous fluids of 3800 mL, along with oral intake of 1500 kcal and 1000 mL of water. Definitive tumor removal was considered essential for the resolution of the underlying condition. The patient was initially referred to our department of gastrointestinal surgery; however, both CT and endoscopic evaluation revealed no evidence of submucosal invasion or malignancy. The patient expressed a strong desire to avoid stoma formation. After thorough multidisciplinary discussion and detailed informed consent, including explanations regarding the potential risks of intraoperative complications such as perforation and bleeding, as well as postoperative complications such as stricture or the possibility of treatment discontinuation, ESD under general anesthesia was selected.

**FIGURE 2:**
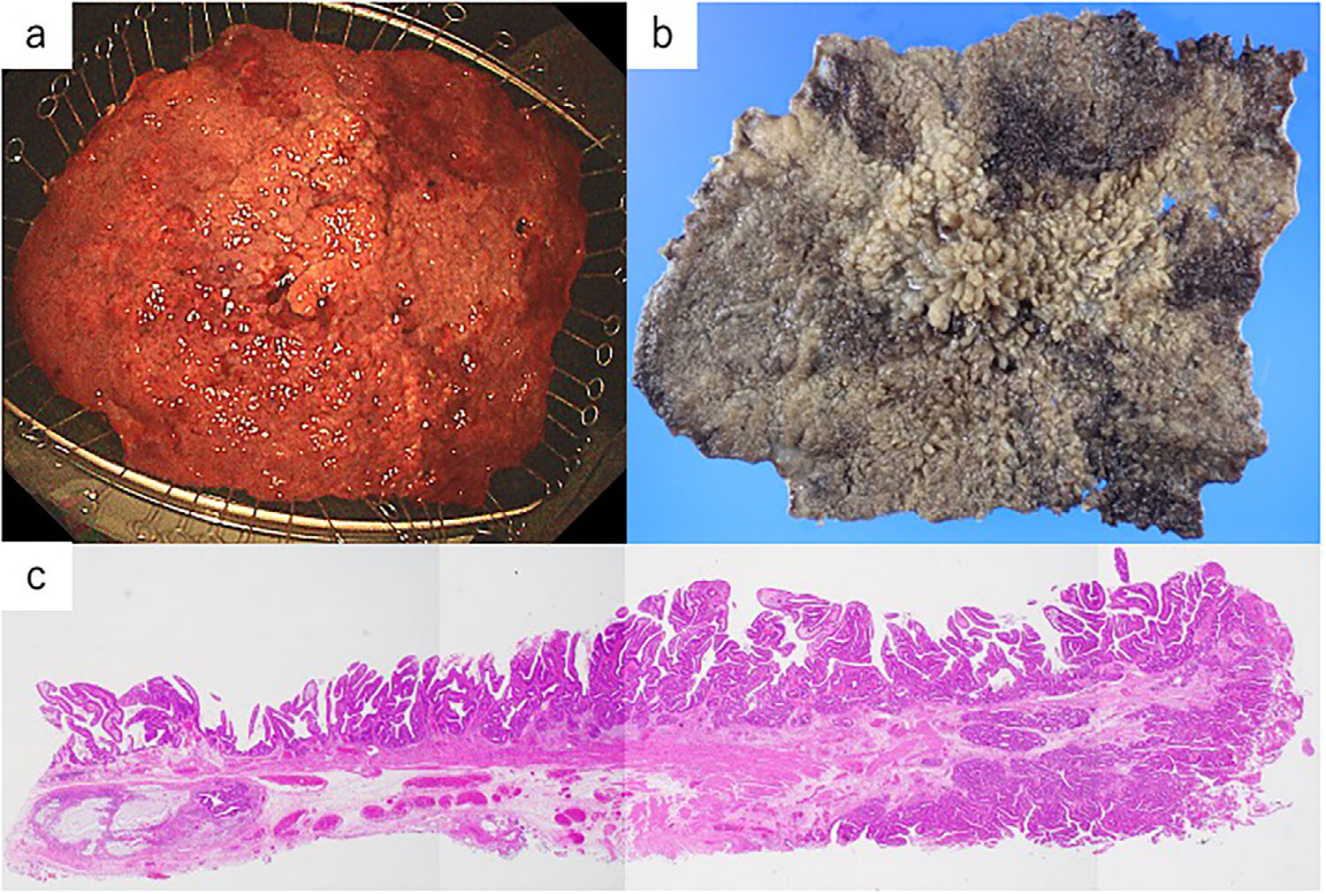
Pathological findings of the resected specimen. (a) Endoscopic image, (b) macroscopic findings, and (c) microscopic findings.

The ESD procedure achieved en bloc resection of a 280 × 240 mm tumor. ESD was performed utilizing the tunnel technique. A total of four submucosal tunnels were established and subsequently interconnected, enabling en bloc resection of the lesion. No major intraoperative complications, such as perforation, occurred. To prevent postoperative stricture, a total of 400 mg of triamcinolone was injected into the base of the ulcer. The total procedure time was 12 h and 33 min. Postoperative labs showed a transient inflammatory response but no anemia or complications. On postoperative day 3, a second‐look colonoscopy revealed no signs of bleeding or perforation, and elemental nutrition was started. Oral feeding began on day 7, and the patient was discharged on postoperative day 10. After ESD, elemental diets and other supportive measures were administered as appropriate, and it took approximately one month for both the laboratory data and diarrhea to stabilize (Table 1). Histopathological analysis demonstrated adenocarcinoma arising in association with adenomatous components, exhibiting predominantly papillary architecture, followed by well‐differentiated tubular (tub1) and moderately differentiated tubular (tub2) structures. The depth of submucosal invasion was measured at 800 µm, with no evidence of lymphovascular invasion. Both the horizontal and vertical resection margins were free of tumor involvement, and curative resection was thus achieved (Figure 2). To prevent stricture, oral prednisolone 30 mg/day (0.5 mg/kg/day) was started on day 7 and tapered by 5 mg every 2 weeks. At 6‐month follow‐up colonoscopy, no recurrence or stricture was observed, and no balloon dilation was required.

## 3 Discussion

EDS is a rare but potentially life‐threatening condition characterized by excessive loss of body fluids and electrolytes, leading to dehydration and electrolyte imbalances [1]. Reported clinical features include occurrence in elderly individuals, long disease duration, rectosigmoid predominance, tumor length ≥10 cm, severe diarrhea, and frequent carcinoma association [5]. Our case demonstrated all features except older age. Traditionally, surgery has been considered the standard therapy, but colectomy often necessitates stoma creation, which significantly reduces quality of life. Recent reports, including those by Kure et al., have shown that ESD can be effective even for lesions exceeding 10 cm, suggesting a growing role for minimally invasive approaches [2].

Our case is notable for the extraordinary size of the tumor, which measured over 28 cm in length and involved the entire circumference of the rectum, making it one of the largest rectal villous tumors reported to date [6, 7]. Despite its considerable size and circumferential spread, we successfully achieved curative en bloc resection via ESD without intraoperative or delayed perforation, and crucially, without the need for surgical colectomy or permanent stoma creation.

Remarkably, the severe secretory diarrhea and associated electrolyte imbalances completely resolved following ESD, confirming the efficacy of tumor resection as a definitive treatment for EDS. Furthermore, through meticulous postoperative management, including the prophylactic administration of systemic steroids and timely second‐look endoscopy, we successfully prevented the development of post‐ESD stricture despite the presence of an extensive mucosal defect.

Circumferential colorectal ESD defects involving ≥90% of the lumen are associated with a high risk of stricture formation [8]. To address this, we adapted preventive strategies from esophageal ESD, where intralesional triamcinolone and systemic corticosteroids have proven effective [9, 10]. The rationale for adapting this approach to the colorectum lies in the shared pathophysiological mechanism of progressive luminal narrowing driven by excessive submucosal fibrosis and cicatricial healing. In our case, the combination of local triamcinolone injection and tapered oral prednisolone successfully prevented stricture. Although evidence for this approach in the colorectum remains limited, our experience suggests it may preserve rectal function and reduce the need for repeated interventions in high‐risk cases.

A comprehensive search of PubMed was conducted regarding electrolyte depletion syndrome using the search terms “electrolyte depletion syndrome” AND “endoscopic submucosal dissection.” Of the three articles retrieved, one was excluded because it did not provide a detailed description of the pathological characteristics of the tumor itself. The remaining articles are summarized in Table 2. Reports have described difficulties in perioperative management, including cases in which ESD was performed in two stages and cases complicated by postoperative stricture. This case represents a successful single‐session ESD for a giant circumferential rectal villous tumor causing EDS, followed by postoperative care that completely prevented stricture formation. The patient's recovery was uneventful, and no recurrence or complication was observed during a six‐month follow‐up period.

**TABLE 2:** Summary of reported cases of electrolyte depletion syndrome treated with endoscopic submucosal dissection (ESD).

Author	Kure et al.	Ohara et al.	Our case
Year	2015	2015	2024
Age	81	66	58
Sex	Male	Female	Male
Chief complaint	Diarrhea	Malaise, anorexia	Diarrhea
Duration of illness (months)	5	1	6
Lesion size (cm)	12.7	24.5	28.0
Location	Rectum	Rectum	Rectum
Histological type	Well‐differentiated adenocarcinoma in a high‐grade adenoma	Adenocarcinoma containing a villous component	Papillary adenocarcinoma with well‐ and moderately‐differentiated tubular adenocarcinoma
Depth of invasion	Mucosa	Mucosa	Submucosa (800 µm)
Lymphovascular invasion	Negative	Negative	Negative
Treatment Difficulties	ESD was performed over two separate days.	Endoscopic balloon dilation (EBD) was required.	—

EBD: Endoscopic balloon dilation.

Careful treatment planning and meticulous postoperative management are essential to achieving favorable outcomes; however, this case demonstrates that ESD can serve as an effective and minimally invasive treatment option for EDS secondary to large rectal villous tumors.

## Author Contributions

**Toshifumi Iida** wrote the article; **Toshifumi Iida** and **Hideyuki Chiba** conceived and designed the study; **Ai Hirohata**, **Akimichi Hayashi**, **Yu Ebisawa**, **Jun Arimoto**, and **Michiko Nakaoka** provided patient management; **Hideyuki Chiba** and **Ken Ohata** provided the final approval of the article.

## Conflicts of Interest

The authors declare no conflicts of interest.

## Supporting information




**Supporting Video 1**: Endoscopic video during ESD procedure.
