# Correction to “Electrolyte Depletion Syndrome due to a 28 cm Rectal Villous Tumor: Successful Endoscopic Resection of One of the Largest Tumors Reported to Date—A Case Report”

**DOI:** 10.1002/deo2.70229

**Published:** 2025-10-25

T. Iida, H. Chiba, A. Hirohata, et al., “Electrolyte Depletion Syndrome due to a 28 cm Rectal Villous Tumor: Successful Endoscopic Resection of One of the Largest Tumors Reported to Date—A Case Report,” *DEN Open* 6 (2026): e70197.

In paragraph 5 of the “Case Report” section, the statement: “The depth of submucosal invasion was measured at 800 µm, with no evidence of lymphovascular invasion. Both the horizontal and vertical resection margins were free of tumor involvement, and curative resection was thus achieved (Figure 2).” was incorrect. The correct description should read: “The depth of submucosal invasion was measured at 800 µm, with no evidence of lymphovascular invasion, and budding grade 1. Although the pathological margin was positive for carcinoma and unclear for adenoma, the resection was considered curative based on endoscopic findings (Figure 2).”

We would like to add the following acknowledgment in relation to the valuable comments provided regarding this lesion.

“Acknowledgments” section, the statement: “The authors wish to thank all the clinical staff who contributed to this paper, and we would like to express our sincere gratitude to Dr. Hiroshi Kawachi (Department of Pathology, Omori Red Cross Hospital, Tokyo, Japan/Department of Pathology, Cancer Institute Hospital, Japanese Foundation for Cancer Research, Tokyo, Japan) for the detailed pathological diagnosis of this case, and to all the pathology staff for their invaluable support.”

We apologize for this error.

